# Improving dynamic stroke risk prediction in non-anticoagulated patients with and without atrial fibrillation: comparing common clinical risk scores and machine learning algorithms

**DOI:** 10.1093/ehjqcco/qcab037

**Published:** 2021-05-17

**Authors:** Gregory Y H Lip, George Tran, Ash Genaidy, Patricia Marroquin, Cara Estes, Jeremy Landsheft

**Affiliations:** Liverpool Centre for Cardiovascular Science, University of Liverpool and Liverpool Heart & Chest Hospital, Liverpool, L14 3PE, UK; Aalborg Thrombosis Research Unit, Department of Clinical Medicine, Aalborg University, 9000 Aalborg, Denmark; Clinical Pharmacy Services, IngenioRX, 450 Headquarters Plaza, 7th Floor East Tower, Morristown, NJ 07960, USA; Clinical Health Economics & Medicaid Clinical Operations, Anthem Inc., 220 Virginia Avenue, Indianapolis, IN 46204, USA; Clinical Health Economics & Medicaid Clinical Operations, Anthem Inc., 220 Virginia Avenue, Indianapolis, IN 46204, USA; Clinical Health Economics & Medicaid Clinical Operations, Anthem Inc., 220 Virginia Avenue, Indianapolis, IN 46204, USA; Clinical Pharmacy Services, IngenioRX, 450 Headquarters Plaza, 7th Floor East Tower, Morristown, NJ 07960, USA

**Keywords:** Atrial fibrillation, Stroke risk prediction, Anticoagulants, Medical/pharmacy claims

## Abstract

**Aims:**

Diversified cardiovascular/non-cardiovascular multi-morbid risk and efficient machine learning algorithms may facilitate improvements in stroke risk prediction, especially in *newly diagnosed non-anticoagulated* atrial fibrillation (AF) patients where initial decision-making on stroke prevention is needed. Therefore the aims of this article are to study common clinical risk assessment for stroke risk prediction in AF/non-AF cohorts together with cardiovascular/ non-cardiovascular multi-morbid conditions; to improve stroke risk prediction using machine learning approaches; and to compare the improved clinical prediction rules for multi-morbid conditions using machine learning algorithms

**Methods and results:**

We used cohort data from two health plans with 6 457 412 males/females contributing 14,188,679 person-years of data. The model inputs consisted of a diversified list of comorbidities/demographic/ temporal exposure variables, with the outcome capturing stroke event incidences. Machine learning algorithms used two parametric and two nonparametric techniques. The best prediction model was derived on the basis of non-linear formulations using machine learning criteria, with the highest c-index was obtained for logistic regression [0.892; 95% confidence interval (CI) 0.886–0.898] with consistency on external validation (0.891; 95% CI 0.882–0.9). These were significantly higher than those based on the conventional stroke risk scores (CHADS2: 0.7488, 95% CI 0.746–0.7516; CHA2DS2-VASc: 0.7801, 95% CI 0.7772–0.7831) and multi-morbid index (0.8508, 95% CI 0.8483–0.8532). The machine learning algorithm had good internal and external calibration and net benefit values.

**Conclusion:**

In this large cohort of newly diagnosed non-anticoagulated AF/non-AF patients, large improvements in stroke risk prediction can be shown with cardiovascular/non-cardiovascular multi-morbid index and a machine learning approach accounting for dynamic changes in risk factors.

## Introduction

Atrial fibrillation (AF) is the commonest cardiac rhythm disorder and confers a five-fold greater risk of stroke.^1^ The risk of stroke is not homogeneous and depends on the presence of various clinical risk factors. The more common and validated stroke risk factors have been used to formulate clinical risk scores for stroke risk stratification, but all clinical scores only have modest predictive value for identifying the ‘high risk’ patients that actually sustain stroke events, with c-indexes (a statistical measure of prediction) of ∼0.6.^1^^,^^2^ More complicated clinical risk scores or the addition of biomarkers will always *statistically* improve on risk prediction, but the absolute difference in c-index is often modest, and clinical utility improvements using decision curve analysis are marginal.^3^^,^^4^ Indeed, the debate is unsettled as to whether adding biomarkers improves the clinical utility of current risk scores, especially since many biomarkers are non-specific and are affected by non-cardiac conditions or are predictive of both thrombotic and bleeding events.^4^^,^^5^

In addition, some complicated clinical risk scores or biomarker-based scores have been derived from highly selected clinical trial cohorts of *anticoagulated* patients, whether on warfarin or a direct oral anticoagulant (DOAC).^6–8^ Improvements in risk prediction are particularly needed, especially in *newly diagnosed non-anticoagulated* patients where decision-making on stroke prevention with oral anticoagulation (OAC) is being considered. Also, many AF patients have pre-existing conditions not accounted for in the existing clinical risk scores that introduce variability that impacts tool performance, for example, valvular heart disease, sleep apnoea, and chronic kidney disease.^9^

In addition, existing clinical risk stratification scores mostly rely on baseline factors and the use of linear terms for the calculation of stroke risk; however, the clinical risk is dynamic and stroke risk changes with age and incident risk factors.^10–12^ With the advent of efficient machine learning technologies, it may be possible to develop complex models, which piece together important clinical and demographic parameters in non-linear formulations, to enhance markedly the performance of such rules. If successful, this has the potential to improve the practice of medicine.^13^^,^^14^ In the absence of selecting important clinical and demographic variables for use in improving the practice of medicine for specific outcomes, the performance of risk models will be at best slightly improved using machine learning techniques relative to conventional methods.^15^^,^^16^ With the above in mind, the application of machine learning techniques should take into account the non-linear effects of prior history of stroke, older age, and multi-morbid conditions.

In the present study, our aim was to perform a comparative assessment of stroke risk prediction in a large non-anticoagulated US cohort via the use of machine learning algorithms compared to the CHADS_2_ and CHA_2_DS_2_-VASc risk scores.^17^^,^^18^ Given the association of stroke with multiple comorbidities beyond the CHADS_2_ and CHA_2_DS_2_-VASc scores, a new multi-morbid index was generated accounting for both cardiovascular and non-cardiovascular clinical history and were comparatively compared to the conventional stroke risk indices and the machine learning algorithms. Model performance attributes are examined in terms of calibration, discrimination, and clinical utility.^19–21^

## Methods

We studied a non-anticoagulated (i.e. not exposed to warfarin or DOAC) population of patients with and without AF. It was drawn from the Commercial plan for the working population and their families (18–64 years) and Medicare plan for the elderly (> 65 years) and those with disabilities (> 18 years) which were used as the primary sources of data. The Medicare plan was derived from the Medicare Advantage and Medicare-Medicaid Advantage (for dual eligible Medicare beneficiaries) enrolment. During the study period (1 January 2016 to 30 June 2020), the targeted population from both plans contributed 2 912 241 males and 3 535 030 females, with follow-up data of 7 656 600 and 6 532 079 person-years, respectively. The population had complete coverage for both medical and pharmacy benefits and was identified from the pharmacy and medical claim databases. IRB approval was not required for the extraction of data from the claim databases; however, compliance with US privacy laws and Company governance is required for use of data.

### Population identification

The process of identifying the non-anticoagulated AF and non-AF populations consisted of several steps: (a) obtain the pharmacy claims for OAC medications (Warfarin or coumadin, eliquis or apixaban, pradaxa or dabigatran etexilate, xarelto or rivaroxaban) together with the member identification; (b) obtain the medical claims for AF members using ICD-10 codes (I480, I481, I4811, I4819, I482, I4820, I4821, I483, I484, I489, I4891, I4892), and extract the associated identification parameters; (c) identify the AF and non-AF members who are not on OACs. Each medication was analysed using both NDC (National Drug Code) and GPI (Generic Product Identifier) codes (see suppl. table S1 for the respective NDC codes for each of the anti-coagulants). This is because NDCs can be ambiguous and many codes exist for a single product, leading to inaccuracies in the dispensing of drugs. Therefore, GPI was used to ensure consistency, with many (from NDC) to one (to GPI) mapping. The medical claims were obtained for primary and secondary AF and non-AF ICD 10 codes.

### Parameter identification

The index condition for an AF target was identified as having two or more medical claims during the period of 1 January 2017 to 30 March 2020. The date of the first claim was the index date. The incidence of stroke outcome was identified as the first event, which occurred after the index date until the end of the study period (30 June 2020). Patients were censored when they had their first stroke, or (as we intended to determine risk without attenuation by anticoagulation use) when AF patients were initiated on OAC.

Comorbid conditions for the AF cohorts were tracked starting from 1 January 2016 until the day prior to the index date. The stroke outcome and comorbid conditions were identified from medical claims using primary and/or secondary diagnoses. Supplementary material online, *Table S2* provides a list of ICD 10 codes for input and output conditions. Gender and age were documented from the medical databases. Age was categorized into four groups (18–54, 55–64, 65–74, and ≥75 years) and was also assessed as a continuous variable.

For non-AF cohorts who are not on anticoagulants, a patient had to have a history of a minimum of 6 months for comorbid conditions upon entry into the study, after which the first incidence of stroke was considered the outcome for these cohorts. As such, the equivalent of AF index date for non-AF cohorts was 6 months after entry into the study.

A comorbid condition or stroke outcome was identified as present (‘1’) or absent (‘0’) and acted as a binary outcome. Gender was treated as a binary variable with ‘1’ for a female and ‘0’ for a male. Recent research has shown that the inclusion of AF duration in stroke risk prediction, which has not been traditionally used in modelling, tends to improve the discrimination validity of the model. In this study, exposure time to AF or non-AF was assessed in two ways: (i) time duration in days from the AF index date or non-AF status date (6 months after entry into the study) to the end of follow-up or benefits; (ii) time duration in days from the last prior stroke case to the AF/non-AF index date. The CHADS_2_ and CHA_2_DS_2_-VASc scores were computed as originally described in the literature.

### Strategy for model development/validation

The large dataset utilized in this study is drawn from the diverse geographical areas covering the US continent. Therefore, the prediction of stroke risk should be coherent to all geographical areas. With this in mind, the training and validation samples were drawn at random from the primary data sources with equal representations of the outcome events. Model development was performed on two-third of the whole population (*training sample*). Validation was performed on the remaining one-third of the data (*validation sample*) once the model developed on the training sample was deemed appropriate on the grounds of clinical meaningfulness and other criteria specific to the predictive algorithm in use.

Model validation was based on calibration (internal and external), discrimination, and clinical utility.^19–22^ Calibration was assessed graphically between the predicted and observed outcomes for the training and validation samples after being subjected to regression smoothing methods such as a locally weighted least squares regression smoother or ‘loess’ algorithm. Discrimination was evaluated using the C-statistic. In addition, external validation was performed using cumulative lift measures. Clinical utility was assessed using decision curve analysis in terms of the net benefit measure at a given probability threshold which reflects the risk at which one is indifferent about the treatment under consideration, with a balance between both sensitivity and specificity in terms of appropriate values.

In this study, we compared the machine learning-based algorithms against the two common stroke risk scores (i.e. CHADS_2_ and CHA_2_DS_2_-VASc scores). We also compared the machine learning-based algorithms against a multi-morbid index.

The multi-morbid index was developed by employing a logistic regression model on the basis of the training sample using main effects with comorbid history, demographic variables (gender, age group as explained above), and other variables (AF status—1 for AF group and 0 for non-AF group via the use of ICD 10 codes as explained before; Medicare status—1 for Medicare plan and 0 for Commercial plan). On the basis of this analysis, the multi-morbid index was constructed as the sum of multi-morbid conditions (2 points for the presence of hypertension or diabetes mellitus; 19 points for the presence of prior stroke history; 1 point for congestive heart failure, vascular disease, valvular disease, coronary artery disease, chronic kidney disease, sleep apnoea, chronic obstructive pulmonary disease, alcohol use or disorders, prior history of major bleeding, inflammatory disease, and lipid disorders; and 0 point for the absence of condition), gender (1 point for female and 0 for male), and age group (0 point for 18–54 years; 4 points for 55–64 years; 8 points for 65–74 years; and 12 points for >75 years). In essence, the multi-morbid index is an enhancement of the CHA_2_DS_2_-VASc score by accounting for non-cardiovascular conditions, additional cardiovascular events, additional weights for stroke history and age groups, as well as an additional age group category from 55 to 64 years). Based on main effect analysis using logistic regression, it was determined that the weight for prior history of stroke should be increased to 19 points and the hypertension and diabetes mellitus weights should be amplified to 2 points. There were 4 points for 55–64 years age group, 8 points for 65–74 years age group, and 12 points for >75 years age group. There was 1 point for each of the following additional conditions: coronary artery disease, valvular disease, sleep apnoea, chronic kidney disease, chronic obstructive pulmonary disease/bronchiectasis, prior history of major bleeding, alcohol use or disorders, inflammatory disease, and lipid disorders. The index ranged from 0 to 44 points.

Four machine learning algorithms were employed, that is, two parametric (logistic regression and neural network) and two non-parametric (decision tree, gradient boosting)^23–25^ (see Supplementary material online, *Table S3* for greater details). The inputs to stroke outcome were the baseline characteristics of comorbid history and demographic variables. In addition, we assessed (i) the temporal characteristics of exposure to AF/non-AF status until the end of follow-up time; and (ii) exposure time from last prior stroke event in comorbid history to AF/non-AF index date. The Statistical Analysis Software Enterprise Miner 15.1 was used to implement these algorithms in a JAVA Web Platform. There was no funding for the study.

## Results

Our final study cohort consisted of 6,457,412 persons [mean (SD) age 43.9 (15.8) years; 55% female] with mean CHADS_2_ and CHA_2_DS_2_-VASc scores of 0.17 (SD 0.50) and 0.83 (SD 0.88), respectively. For the AF patients, there were 128,047 persons [mean (SD) age 69.4 (12.4) years; 44% female] with mean CHADS_2_ and CHA_2_DS_2_-VASc scores of 1.08 (SD 1.23) and 2.28 (SD 1.72), respectively). The non-AF cohort included 6,329,365 persons [mean (SD) age 43.4 (15.4) years; 55% female] with mean CHADS_2_ and CHA_2_DS_2_-VASc scores of 0.15 (SD 0.45) and 0.80 (SD 0.83), respectively.

For the training and validation samples in AF cohorts, the prevalence of hypertension was the highest (34.5%) followed by lipid disorders (28.9%), coronary artery disease (15.9%), congestive heart failure (13.7%), valvular disease (12.8%), then chronic obstructive pulmonary disease (12.6%). For the training and validation samples in non-AF cohorts, the comorbidities are summarized in *Table 1*. In general, the non-AF cohort had significantly less comorbid conditions than AF cohorts. The entirety of AF and non-AF cohorts was not taking anticoagulants.

**Table 1:** Baseline characteristics and common stroke risk scores for the training and validation cohorts used in development and validation of stroke risk prediction algorithms

Baseline characteristics	Training		Validation	
	AF cohort	Non-AF cohort	AF cohort	Non-AF cohort
Gender				
Females	37 166 (43.5)	2 319 398 (55.0)	18 581 (43.6)	1 159 958 (55.0)
Males	48 221 (56.5)	1 900 372 (45.0)	24 079 (56.4)	949 637 (45.0)
Age (years), mean (SD)	69.4 (12.4)	43.4 (15.4)	69.5 (12.4)	43.4 (15.4)
Comorbid history				
Congestive heart failure	11 794 (13.8)	25 196 (0.6)	5809 (13.6)	12 706 (0.6)
Hypertension	29 406 (34.4)	287 805 (6.8)	14 710 (34.5)	143 973 (6.8)
Diabetes mellitus	9621 (11.3)	138 542 (3.3)	4693 (11.0)	69 157 (3.3)
Stroke	5191 (6.1)	20 134 (0.5)	2541 (6.0)	10 184 (0.5)
Vascular disease	9713 (11.4)	43 103 (1.0)	4786 (11.2)	21651 (1.0)
Valvular disease	10 925 (12.8)	40 835 (1.0)	5466 (12.8)	20 408 (1.0)
Coronary artery disease	13 681 (16.0)	73 927 (1.8)	6742 (15.8)	37 055 (1.8)
Chronic sleep apnoea	5862 (6.9)	168 053 (4.0)	2959 (6.9)	83 557 (4.0)
Chronic kidney disease	8035 (9.4)	49 140 (1.2)	3903 (9.1)	24 233 (1.1)
Chronic pulmonary obstructive disease/bronchictasis	10814 (12.7)	104 430 (2.5)	5303 (12.4)	52260 (2.5)
Major bleeding	5398 (6.3)	50 757 (1.2)	2485 (5.8)	25277 (1.2)
Alcohol use	1175 (1.4)	32 224 (0.8)	600 (1.4)	15823 (0.8)
Alcohol disorders	369 (0.4)	3886 (0.1)	179 (0.4)	1889 (0.1)
Inflammatory diseases	3587 (4.2)	58 683 (1.4)	1739 (4.1)	29 148 (1.4)
Lipid disorders	24 776 (29.0)	552 165 (13.1)	12272 (28.8)	276356 (13.1)
CHADS_2_				
0	34 928 (40.9)	3 728 127 (88.3)	17 292 (40.5)	1 863 678 (88.3)
1	26 731 (31.3)	385 001 (9.1)	13600 (31.9)	192 552 (9.1)
2	12 193 (14.3)	86 216 (2.0)	6128 (14.4)	43 107 (2.0)
3	6893 (8.1)	16 055 (0.4)	3433 (8.0)	8099 (0.4)
4	3069 (3.6)	3575 (0.1)	1450 (3.4)	1766 (0.08)
5	1229 (1.4)	706 (0.02)	605 (1.4)	349 (0.02)
6	344 (0.4)	90 (0.002)	152 (0.4)	44 (0.002)
CHA_2_DS_2_-VASc				
0	12 998 (15.2)	1583 140 (37.4)	6382 (15.0)	791 561 (37.5)
1	18 660 (21.9)	2 157 098 (51.1)	9285 (21.8)	1 078 028 (51.1)
2	19 372 (22.7)	295 969 (7.0)	9859 (23.1)	148 304 (7.0)
3	16 226 (19.0)	123 012 (2.9)	8194 (19.2)	61 149 (2.9)
4	8447 (9.9)	44 523 (1.1)	4219 (9.9)	22 523 (1.1)
5	5210 (6.1)	11 842 (0.3)	2546 (6.0)	5966 (0.3)
6	2732 (3.2)	3186 (0.1)	1342 (3.1)	1556 (0.1)
7	1199 (1.4)	816 (0.02)	582 (1.4)	417 (0.02)
8	446 (0.5)	155 (0.004)	212 (0.5)	82 (0.004)
9	97 (0.1)	29 (0.001)	39 (0.1)	9 (0.0004)

Values are numbers (%) unless stated otherwise.

### Comparative assessment of stroke risk indices

*Table 2* shows the incidence rates in cases per 100 person-years for the CHADS_2_ and CHA_2_DS_2_-VASc scores as well as the multi-morbid index scores. The results are provided for low-, medium-, and high-risk scores for both the AF and non-AF cohorts. In general, the CHADS_2_ scores had the highest incidence rates followed by the CHA_2_DS_2_-VASc scores, then the multi-morbid index scores for all three risk levels and AF/non-AF cohorts. The incidence rates for the AF cohorts were significantly much higher than those for the non-AF cohorts at the 5% level.

**Table 2:** Stroke outcomes for three stroke risk indices. Values are cohort size, number of incidence cases, and incidence rate in cases per 100 person-years (95% CI)

		AF cohort			Non-AF cohort		
Stroke risk index		Cohort size	Stroke outcome		Cohort size	Stroke outcome	
			No. of events	Incidence rate ‘cases/100 person-years’ (95% CI)		No. of events	Incidence rate ‘cases/100 person-years’ (95% CI)
CHADS_2_							
	Low risk (0)	52 220	4566	5.12 (4.97–5.27)	5 591 805	35497	0.30 (0.29–0.30)
	Medium risk (1)	40 331	5064	6.17 (6.00–6.34)	577 553	20130	1.38 (1.36–1.40)
	High risk (2–6)	35 496	8636	8.87 (8.68–9.06)	160 007	23899	5.19 (5.13–5.26)
CHA_2_DS_2_-VASc							
	Low risk (0)	19 380	1172	3.59 (3.38–3.79)	2 374 701	11737	0.23 (0.22–0.23)
	Medium risk (1)	27 945	2461	4.78 (4.59–4.97)	3 235 126	20794	0.30 (0.30–0.31)
	High risk (2–9)	80 722	14 633	7.93 (7.80–8.06)	719 538	46995	2.54 (2.52–2.56)
Multi-morbid index							
	Low risk (0)	6400	256	2.46 (2.16–2.76)	1796173	5391	0.14 (0.14–0.14)
	Medium risk (1–4)	16809	1113	3.79 (3.57–4.01)	3241527	16402	0.24 (0.24–0.24)
	High risk (5–44)	104838	16897	7.38 (7.27–7.49)	1291665	57733	1.86 (1.84–1.88)

**Figure 1:**
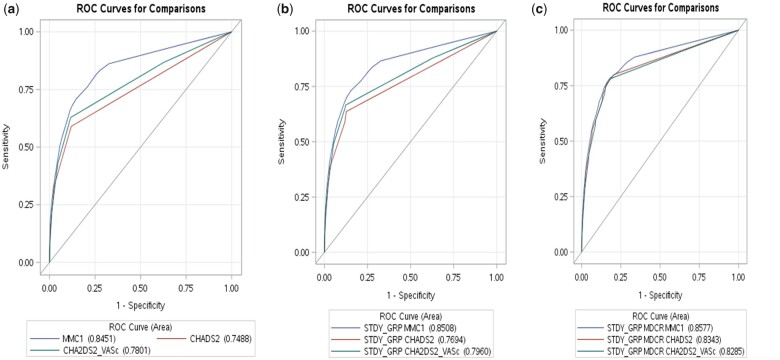
External validation for three sets of three clinical rule-based models: (*A*) Set 1: multi-morbid index ‘MMC1’ (C index 0.8451, 95% CI 0.8427–0.8476), CHADS_2_ (C index 0.7488, 95% CI 0.746–0.7516), CHA_2_DS_2_VASc (C index 0.7801, 95% CI 0.7772–0.7831); (*B*) Set 2: multi-morbid index/AF status (C index 0.8508, 95%CI 0.8483–0.8532), CHADS_2_/AF status (C index 0.7694, 95% CI 0.7667–0.7722), CHA_2_DS_2_VASc/AF status (C index 0.796, 95% CI 0.7931–0.7989); (*C*) Set 3: multi-morbid index/AF status/Medicare status (C index 0.8577, 95% CI 0.8553–0.86), CHADS_2_/AF status/Medicare status (C index 0.8343, 95% CI 0.8319–0.8368), CHA_2_DS_2_VASc/AF status/Medicare status (C index 0.8285, 95% CI 0.8259–0.8312).

*Table 3* shows a summary of three sets of models based on the three types of stroke risk indices for the training samples. Set 1 is only based on the stroke risk index and sets 2 and 3 based on (i) the stroke risk index/AF group status and (ii) stroke risk index/AF group status/Medicare group status, respectively. The C indices were higher for set 3, followed by set 2, then set 1. *Figure 1* shows similar results based on external validation.

**Table 3:** CHADS_2_-, CHA_2_DS_2_VASc-, and multi-morbid index-based stroke risk prediction models: C-statistic, variables, and corresponding odds ratios (95% CI)/significance levels

Model description	Model no.	C-statistic	Variable	Odds ratio (95% CI)	Significance level
CHADS_2_-based	1	0.749	CHADS_2_	3.55 (3.53–3.58)	<0.0001
	2	0.770	AF status	3.17 (3.14–3.19)	<0.0001
			CHADS_2_	3.14 (3.06–3.22)	<0.0001
	3	0.835	Medicare status	5.87 (5.76–5.98)	<0.0001
			AF status	2.36 (2.30–2.42)	<0.0001
			CHADS_2_	2.18 (2.16–2.20)	<0.0001
CHA_2_DS_2_-VASc-based	4	0.781	CHA_2_DS_2_-VASc	2.47 (2.46–2.48)	<0.0001
	5	0.797	AF status	2.94 (2.86–3.01)	<0.0001
			CHA_2_DS_2_**-**VASc	2.28 (2.27–2.30)	<0.0001
	6	0.830	Medicare status	4.67 (4.57–.77)	<0.0001
			AF status	2.67 (2.60–2.73)	<0.0001
			CHA_2_DS_2_-VASc	1.69 (1.68–1.70)	<0.0001
Multi-morbid index-based	7	0.845	Multi-morbid index	1.21 (1.21–1.21)	<0.0001
	8	0.851	AF status	2.73 (2.66–2.80)	<0.0001
			Multi-morbid index	1.20 (1.20–1.20)	<0.0001
	9	0.858	Medicare status	2.88 (2.82–2.94)	<0.0001
			AF status	2.46 (2.40–2.52)	<0.0001
			Multi-morbid index	1.16 (1.15–1.17)	<0.0001

**Figure 2:**
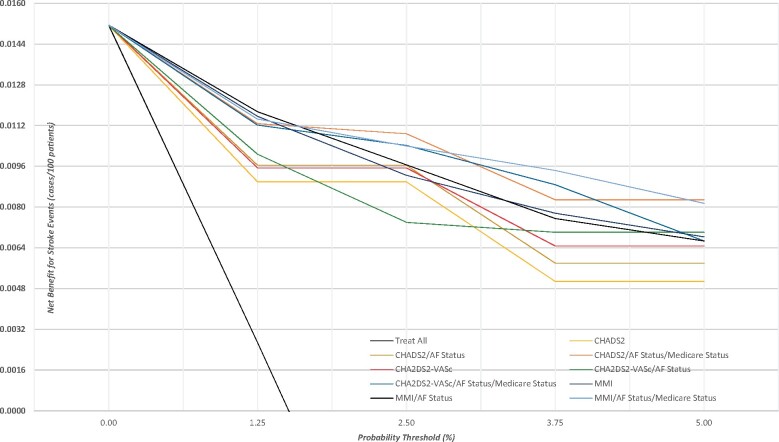
Clinical utility for stroke risk-based models using validation samples. AF, atrial fibrillation; MMI, multi-morbidity index.

*Figure 2* shows the clinical utility of three sets of stroke risk-based models. In general, any of the models has a higher net benefit than the ‘treat all’ and ‘treat none’ strategies, therefore, these models are clinically useful at designated probability thresholds. The multi-morbid index-based models have the highest net benefit in terms of stroke cases per 100 patients adjusted for any false positives at a given probability threshold.

### Machine learning algorithms relative to stroke risk scores

Based on the training samples, the highest c-index was obtained for logistic regression at 0.892 [95% confidence interval (CI) 0.886–0.898]. This was followed by gradient boosting (C-index 0.886, 95% CI 0.88–0.891), decision tree (C-index 0.883, 95% CI 0.877–0.889), and finally neural network (C-index 0.86, 95% CI 0.852–0.868). As such, the non-linear formulations of machine learning incrementally outperformed the aforementioned stroke risk indices.

**Figure 3:**
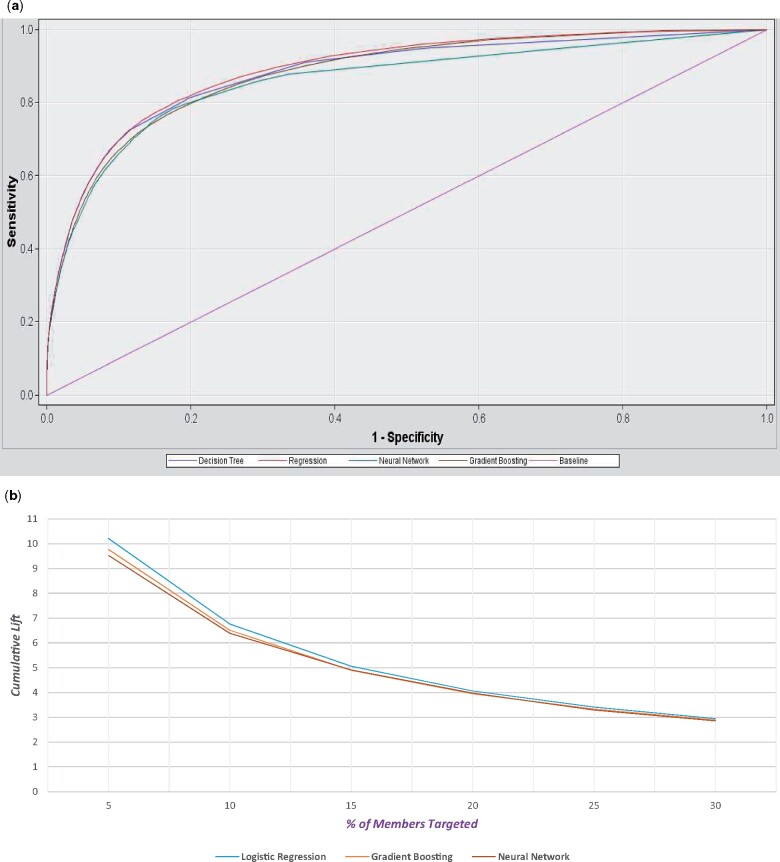
External validation of machine learning algorithms—(*A*) ROC for decision tree (blue); logistic regression (red), neural network (dark grey); gradient boosting decision tree (brown), baseline (purple); (*B*) cumulative lifts for logistic regression, gradient boosting, and neural network.

External validation of the machine learning algorithms showed similar performance (*Figure 3*). The area under the curve values were (*Figure 3A*): logistic regression (C index 0.891, 95% CI 0.882–0.9), gradient boosting (C index 0.881, 95% CI 0.872–0.89), decision tree (C index 0.881, 95% CI 0.872–0.89), and neural network (C index 0.859, 95% CI 0.848–0.87).

*Figure 3B* demonstrates that the logistic regression algorithm outperformed the gradient boosting and neural network algorithms in external validation in terms of cumulative lift. While the area under the curve provides a measure of true positives vs. false positives, the cumulative lift demonstrates a snapshot of the ratio of the percentage of patients with stroke events reached during a treatment campaign to the percentage of patients targeted. The cumulative lifts for 5% of member targeted were 10.21, 9.61, and 9.21, respectively for the logistic regression, gradient boosting, and neural network algorithms. Thus, 51.1% or 16 639 events (predicted using logistic regression), 48.8% or 15 913 events (predicted using gradient boosting), and 47.6% or 15 525 events (predicted using neural network) of patients with stroke events were reached when 5% of members were targeted.

Supplementary material online, *Figure S1* indicates the incremental improvement of the machine learning-based logistic regression algorithm over the full multi-morbid index-based model in terms of clinical utility. At a probability threshold of 3.75%, the machine learning algorithm produced a net benefit of 1.0 true stroke events relative to the 0.94 stroke event achieved using the full multi-morbid-index-based model. At this probability threshold, both models are much more clinically useful relative to the all treatment strategy; yet the machine learning algorithm provided a total of 14 441 true stroke events relative to the 12 642 events using the full-multi-morbid index-based model.

Supplementary material online, *Figure S2* shows the importance of the multi-morbid index, AF status, and Medicare status as variables in the machine learning-based algorithms. For simple indices, one can opt for the full multi-morbid index-based model; however, complex formulations such as machine learning-based algorithms afford us the opportunity to capture more events for severe clinical outcomes such as stroke.

## Discussion

In this large contemporary cohort of newly diagnosed non-anticoagulated patients with AF/non-AF, our principal findings are the demonstration of improved stroke risk prediction using cardiovascular and non-cardiovascular multi-morbid conditions and machine learning approaches, compared to the two conventional clinical risk scores, the CHADS_2_ and CHA_2_DS_2_-VASc scores. Indeed, the highest c-index was obtained for logistic regression (0.892), with consistency on external validation (0.891).

The present study based on a large US cohort validates the clinical meaningfulness of the CHADS_2_ and CHA_2_DS_2_-VASc scores^17^^,^^18^ developed on smaller sample sizes approximately 20 and 10 years ago, respectively, which showed good calibration even in this contemporary cohort. The discriminant validity of these tools showed moderately good c-indexes of >0.7. In an independent PCORI systematic review and evidence appraisal, the CHADS_2_, CHA_2_DS_2_-VASc, and ABC-stroke scores had the best prediction for stroke events.^2^ While the CHADS_2_ was simple, its use has been super-ceded by the CHA_2_DS_2_-VASc score in many contemporary guidelines, given the default has shifted to offer stroke prevention (which Is OAC) unless the patient is ‘low risk’ (and the CHA_2_DS_2_-VASc score could help initially identify those low-risk patients).^3^ Nonetheless, both clinical risk scores are simplifications, have modest predictive value, and do not account for the dynamic nature of risk.

This study also constructed a multi-morbid index consisting of cardiovascular and non-cardiovascular comorbid conditions. In general, it had more discriminatory power than the two conventional clinical scores as it explained more of the variance in stroke outcome. Additionally, the multi-morbid index, which is a modification of the CHA_2_DS_2_-VASc score in terms of more comorbid conditions, changed weights for prior stroke, hypertension, and diabetes mellitus, and lowered the age threshold to ≥55 years with more weights for different age groups, provided the best performance from among the stroke risk indices.

In the present study, we gain an additional improvement in stroke risk prediction in AF/non-AF cohorts from the use of various comorbid conditions and their synergistic effects with age, gender, and temporal exposure using claims information. This considerable gain was achieved due to the non-linear formulations of aforementioned variables via machine learning algorithms, leading to a c-index with logistic regression of 0.892, with consistency on external validation (0.891), as well as resulting in net benefit values better than the ‘treat all’ strategy or current clinical risk scores. In addition, at the 3.75% risk threshold, the machine learning model showed better clinical utility in comparison to the three-stroke risk indices across all levels of probability thresholds.

The inclusion of AF exposure in terms of the cumulative temporal exposure not taking anticoagulant medication from the index date to the end of follow-up or benefits as well as from the last prior stroke event to the AF/non-AF index date added considerable explanation of the variability in stroke risk prediction. This emphasizes the importance of temporal exposure of AF status in stroke risk prediction, given the dynamic nature of stroke risk and how risk can be influenced by incident risk factors, ageing, and AF progression.^10–12^^,^^26^ The relationship among prior clinical history as measured by the multi-morbid index and demographic variables showed considerable improvement in stroke risk prediction due to the non-linear formulations reported by using machine learning algorithms in terms of two-factor interactions.^27^

### Practical implications

From clinical risk, one can predict stroke risk to a reasonable degree via the diversified comorbid history and age/gender information and simple clinical risk scores. The present study in a contemporary cohort expanded the utility of stroke risk prediction for patients with additional comorbidities and using machine learning techniques. The ability to improve predictive precision, which was also translated into improved clinical utility could facilitate dynamic stroke risk assessments. The possible incorporation of machine learning risk prediction into Apps and smart mobile health (mHealth) technology would enable ‘real time’ dynamic assessments of stroke (and possibly bleeding) risk. Indeed, the AF patient pathway (or ‘patient journey’) would require risk reassessment(s) at intervals, when not on antithrombotic therapy (e.g. when newly diagnosed), and while on aspirin (e.g. with background vascular disease) and post-OAC (whether on warfarin or DOAC). Machine learning could adapt to these treatment changes over time, as well as incident risk factors, and is the subject of ongoing analyses.

The potential opportunities here are illustrated by our Mobile Health (mHealth) technology to improve optimization of integrated care in patients with Atrial Fibrillation App program (mAFA) which investigated mHealth technology for improved screening and integrated care in patients with AF, facilitating early diagnosis, dynamic (re)assessments of risk profiles, and holistic AF management.^28^ In our prospective randomized clinical trial, this integrated care approach significantly reduced the composite outcome of ‘ischaemic stroke/systemic thromboembolism, death, and rehospitalization’ compared with usual care.^29^ Prospective dynamic monitoring and re-assessment of bleeding risk using the HAS-BLED score was associated with less major bleeding events, a reduction in modifiable bleeding risk factors, and increased OAC uptake; in contrast, bleeding rates were higher and OAC use decreased by 25% in the ‘usual care’ arm, when the baseline was compared to 12 months. Incorporation of a dynamic machine learning model into our mHealth technology would facilitate ‘real time’ assessment of stroke risk, facilitating mitigation of modifiable risk factors (e.g. blood pressure control).

### Limitations

Our study is limited by its observational design, but currently represents the largest contemporary cohort of non-anticoagulated ‘real world’ patients for the assessment of stroke risk and comparisons of risk prediction models. As with observational cohorts, the possibility of residual confounding remains.^30^

## Data availability

Data are available as presented in the article. According to US laws and corporate agreements, our own approvals to use the Anthem and IngenioRx data sources for the current study do not allow us to distribute or make patient data directly available to other parties.

**Conflict of interest**: The authors have no conflict of interest for the published work.

## Supplementary Material

qcab037_Supplementary_Data
