# Supplementary material for: Improving dynamic stroke risk prediction in non-anticoagulated patients with and without atrial fibrillation: comparing common clinical risk scores and machine learning algorithms
Source: Eur Heart J Qual Care Clin Outcomes. 2021 May 17;8(5):548–56. doi: 10.1093/ehjqcco/qcab037 (PMC9382661; doi:10.1093/ehjqcco/qcab037)
Supplement: qcab037_Supplementary_Data [file qcab037_supplementary_data.docx]

**Suppl. table S1** Details of NDC codes for anticoagulants

| **Medication / Healthcare Service Name** | **NDC Code Values** |
| --- | --- |
| Warfarin | '00182267101','00182267110','00182267189','00339653712','00615454729','00615454753','00615454763','00781035207', |
|  | '51079090820','59772035204','59772035207','59772035208','00056016901','00056016970','00056016975','00056016990', |
|  | '00093171201','00378880101','00378880110','00406205201','00406205210','00555083102','00555083105','00832121100', |
|  | '00832121101','00832121110','00832121189','15330010001','15330010010','21695067230','23490647801','23490647802', |
|  | '23490647803','31722032701','31722032710','35356054090','35356058230','35356058260','35356058290','42549049730', |
|  | '43063047130','43063065530','43353002830','43353002860','43353049330','43353049360','43353058430','43353058460', |
|  | '51138005430','51138017930','51138019530', |
|  | '51138048210','51138048230','51672402701','51672402703','51672402707','52959092430','54569444300','54569444301', |
|  | '54569622500','54569622501','54868212800','54868212801','54868212802','54868212803','54868434900','54868434901', |
|  | '54868434902','54868434903','54868434905','55045288001','55048085630','55289034030','55887026430','55887026460', |
|  | '55887026482','55887026490','57237011901','57237011999','58118402703','58118402706','58118402709','58864035715', |
|  | '58864077315','58864077330','60429078401','60429078410','60429078415','60429078430','60429078445','60429078477', |
|  | '60760003130','63629401701','63629401702','63629401703','63629401704','63629401705','65162076110','65162076111', |
|  | '66105051810','66267028530','66267062900','66336024920','66336024930','66336024960','68084014677','68115035930', |
|  | '68115035960','68115035990','68258910401','68382005201','68382005210','76282032701','76282032710','00056017030', |
|  | '00182267201','00182267210','00182267289','00339653812','00615150929','00615150953','00615150963','51079090920', |
|  | '54569015801','55289014397','59772036304','59772036307','59772036308','00056017001','00056017070','00056017075', |
|  | '00056017090','00093171301','00093171310','00378880201','00378880210','00406205301','00406205310','00555086902', |
|  | '00555086905','00781036307','00832121200','00832121201','00832121210','00832121289','15330010101','15330010110', |
|  | '21695067330','21695067360','23490648001','23490648002','23490648003','31722032801','31722032810','33358036000', |
|  | '35356057130','35356057160','35356057190','42549049630','43353002130','43353002135','43353002140','43353002145', |
|  | '43353002150','43353002153','43353002155','43353002161','51138005530','51138018030','51138018060','51138019630', |
|  | '51138019660','51138048330','51672402801','51672402803','51672402807','52959092530','53217021930','53217021990', |
|  | '54569015800','54569622400','54868082200','54868212900','54868212901','54868212902','54868212903','54868442200', |
|  | '54868442201','54868442202','54868442203','54868442204','54868442205','55045290208','55048086130','55887092690', |
|  | '57237012001','57237012099','58118402803','58864003014','58864003030','58864030114','58864087930','60429078501', |
|  | '60429078510','60429078515','60429078530','60429078535','60429078540','60429078545','60429078560','60429078577', |
|  | '60429078590','60760004030','62584098401','62584098411','62584098477','63629412201','63629412202','63629412203', |
|  | '63629412204','63629412205','63629412206','63739036001','63739036003','63739036015','65162076210','65162076211', |
|  | '66116046930','66267063600','66336025020','66336025030','66336025090','67544031815','67544031830','67544031835', |
|  | '67544031840','67544031845','67544031850','67544031853','67544031855','67544031860','67544031861','67544031870', |
|  | '67544040115','67544040130','67544040135','67544040140','67544040145','67544040150','67544040153','67544040155', |
|  | '67544040160','67544040161','67544040170','68115009330','68115039930','68258102601','68382005301','68382005310', |
|  | '76282032801','76282032810','00056017630','00182267301','00182267310','00182267389','00339653912','00615151029', |
|  | '51079091020','59772036404','59772036407','59772036408','62584098601','00056017601','00056017670','00056017675', |
|  | '00056017690','00093171401','00093171410','00378882501','00378882510','00406206401','00406206410','00555083202', |
|  | '00555083205','00781036407','00832121301','00832121310','00832121389','12280031230','12280031260','12280031290', |
|  | '15330010201','15330010210','16590034030','16590034060','16590034090','21695067430','23490648101','23490648102', |
|  | '23490648103','31722032901','31722032910','33261099730','33261099760','33261099790','35356039730','35356039760', |
|  | '35356039790','43353002930','43353002935','43353002940','43353002945','43353002953','43353002960','43353014230', |
|  | '43353014235','43353014240','43353014245','43353014253','43353014260','49999041130','51138005630','51138018130', |
|  | '51138019730','51138048410','51138048430','51138048445','51672402901','51672402903','51672402907','54569021201', |
|  | '54569021202','54569586800','54569586801','54868215400','54868215401','54868215402','54868215403','54868440000', |
|  | '54868440001','54868440002','54868440003','54868440004','55048085730','55887057730','55887057760','55887057790', |
|  | '57237012101','57237012199','58118402903','58118402906','58118402909','58864003530','60429078601','60429078610', |
|  | '60429078615','60429078630','60429078645','60429078677','60760003330','63187074530','63629317701','63629317702', |
|  | '63739036101','63739036103','63739036110','63739036115','65162076310','65162076311','66105017610','66267063000', |
|  | '66336025130','66336025160','67544019530','67544019540','67544019545','67544019553','67544019560','68084002701', |
|  | '68084002711','68084002777','68115009230','68258910101','68382006401','68382006410','76282032901','76282032910', |
|  | '00182267401','00182267489','00339654012','00615454829','00615454853','00615454863','00781036607','51079091120', |
|  | '59772036607','00056018801','00056018870','00056018875','00056018890','00093171501','00378880301','00378880310', |
|  | '00406205401','00406205410','00555092502','00832121400','00832121401','00832121410','00832121489','15330026601', |
|  | '21695067530','31722033001','31722033010','33261099830','33261099860','33261099890','35356090630','35356090690', |
|  | '43353003030','43353003060','43353008930','43353008960','43353049230','43353049260','43683011830','51138005730', |
|  | '51138018230','51138019830','51138048510','51138048530','51672403001','51672403003','51672403007','53217023530', |
|  | '54569639400','54868406300','54868406301','54868487100','54868487101','54868487102','54868487103','54868542500', |
|  | '57237012201','57237012299','58118403003','58118403006','58118403009','60429078701','60429078710','60429078715', |
|  | '60429078730','60429078745','60429078777','60760004130','63739036201','63739036203','63739036210','63739036215', |
|  | '65162076410','65162076411','66267063100','68084014777','68382005401','68382005410','71335058001','71335058002', |
|  | '76282033001','76282033010','00182267501','00182267589','00339654112','00615454929','00615454953','00615454963', |
|  | '00781036907','51079091220','59772036907','59772036908','00056016801','00056016870','00056016875','00056016890', |
|  | '00093171601','00378880401','00378880410','00406205501','00406205510','00555087402','00555087405','00832121500', |
|  | '00832121501','00832121510','00832121589','15330026701','21695093930','23490648201','23490648202','23490648203', |
|  | '31722033101','31722033110','33261099000','33261099030','33261099060','33261099090','43063021830','43353003330', |
|  | '43353004930','43353004960','49999092310','49999092330','49999092360','49999092390','51138005830','51138018330', |
|  | '51138019930','51138048610','51138048630','51672403101','51672403103','51672403107','54569586900','54868082500', |
|  | '54868339900','54868339901','54868440200','54868440201','54868440202','54868440203','55048085830','55887046430', |
|  | '55887046460','55887046490','57237012301','57237012399','58016008300','58016008330','58016008360','58016008390', |
|  | '58118403103','58118403106','58118403109','60429078801','60429078810','60429078815','60429078830','60429078845', |
|  | '60429078877','60760004330','60760070630','63629474801','63739036301','63739036303','63739036310','63739036315', |
|  | '65162076510','65162076511','66267063200','67544019430','67544019460','68084014877','68258606703','68382005501', |
|  | '68382005510','71335045201','71335045202','71335045203','71335045204','76282033101','76282033110','00056017230', |
|  | '00182267601','00182267610','00182267689','00339654212','00615151229','00781037707','51079091320','54569015901', |
|  | '55175538003','55289028601','55289028697','59772037704','59772037707','59772037708','60346038125','62584094477', |
|  | '00056017201','00056017270','00056017275','00056017290','00093172110','00378880501','00378880510','00406205601', |
|  | '00406205610','00555083302','00555083305','00832121600','00832121601','00832121610','00832121689','15330026801', |
|  | '15330026810','16590034130','16590034160','16590034190','21695067730','23490648301','23490648302','23490648303', |
|  | '31722033201','31722033210','33261035707','33261035714','33261035720','33261035721','33261035728','33261035730', |
|  | '33261035760','33261035790','33358036130','43063017614','43063017630','43353002311','43353002330','43353002335', |
|  | '43353002338','43353002340','43353002344','43353002345','43353002346','43353002350','43353002353','43353002365', |
|  | '43353002370','43353005011','43353005015','43353005020','43353005021','43353005025','43353005028','43353005030', |
|  | '43353005035','43353005038','43353005040','43353005044','43353005045','43353005046','43353005047','43353005050', |
|  | '43353005053','43353005055','43353005059','43353005060','43353005061','43353005065','43353005068','43353005070', |
|  | '43353005078','43683011730','49999009330','49999057600','49999057610','49999057620','49999057630','49999057660', |
|  | '49999057690','50090002800','51138005930','51138018430','51138020030','51138048710','51138048720','51138048730', |
|  | '51138048775','51672403203','51672403207','52959092630','54569015900','54569493400','54569493401','54569493402', |
|  | '54868125900','54868125901','54868125902','54868125903','54868125904','54868125905','54868125906','54868125907', |
|  | '54868428600','54868428601','54868428602','54868428603','54868428604','54868428605','54868520700','54868520701', |
|  | '55045288108','55048085930','55048088030','55289028614','55289028630','55289028650','55289077314','55289077330', |
|  | '55289077360','55289077390','55887057810','55887057830','55887057860','55887057886','55887057890','57237012401', |
|  | '57237012499','58118403203','58517036030','58864022314','58864022330','58864069814','58864069830','60429078901', |
|  | '60429078910','60429078915','60429078920','60429078925','60429078930','60429078935','60429078940','60429078945', |
|  | '60429078950','60429078960','60429078975','60429078977','60429078990','60760003430','61919034130','62584099401', |
|  | '62584099411','62584099477','63187067410','63187067460','63187067490','63629254801','63629254802','63739036401', |
|  | '63739036403','63739036410','63739036415','65162076610','65162076611','65243027403','66105011010','66116047030', |
|  | '66267026830','66267063300','66336025214','66336025230','66336025260','66336025290','67544005215','67544005220', |
|  | '67544005225','67544005228','67544005230','67544005235','67544005238','67544005240','67544005245','67544005250', |
|  | '67544005253','67544005255','67544005257','67544005260','67544005261','67544005265','67544005268','67544005270', |
|  | '67544005278','68115009430','68115052730','68115052760','68115052790','68115065900','68258102701','68258910201', |
|  | '68382005601','68382005610','68382005616','71335024301','71335024302','71335024303','71335024304','71335024305', |
|  | '71335024306','71610017330','71610017335','71610017340','71610017345','76282033201','76282033210','00182267701', |
|  | '00182267789','00339654312','00615455029','00781038107','51079091420','59772038107','00056018901','00056018970', |
|  | '00056018975','00056018990','00093171801','00378880601','00378880610','00406205701','00555092602','00832121700', |
|  | '00832121701','00832121710','00832121789','15330010601','33261099930','43353058730','51138006030','51138018530', |
|  | '51138020130','51138048810','51138048830','51672403301','51672403303','54569631200','54868121600','54868487300', |
|  | '54868487301','54868487302','54868487303','54868487304','54868525500','54868525501','55700000530','55700000560', |
|  | '55700000590','57237012501','57237012599','58118403303','58118403306','58118403309','60429079001','60429079010', |
|  | '60429079015','60429079030','60429079045','60429079077','65162076710','65162076711','66105052110','66267063400', |
|  | '67544007030','68382005701','76282033301','76282033310','00182267801','00182267889','00339654412','00555083405', |
|  | '00615455129','00781038607','51079091520','59772038607','00056017301','00056017370','00056017375','00093171901', |
|  | '00093172301','00378887501','00378887510','00406205801','00555083402','00832121800','00832121801','00832121850', |
|  | '00832121889','15330010701','21695094030','23490648401','23490648402','23490648403','31722033401','43353005330', |
|  | '43353049430','43353057930','49999082900','51138006130','51138018630','51138020230','51138048930','51672403401', |
|  | '51672403403','53217001830','53217001860','53217001890','54569631300','54569631301','54868225200','54868225201', |
|  | '54868495000','54868495001','54868495002','57237012601','57237012699','58118403403','58118403406','58118403409', |
|  | '60429079101','60429079115','60429079130','60429079145','60429079177','63629441701','63629441702','63629441703', |
|  | '65162076810','65162076811','66267063500','66336082530','68258909701','68382005801','76282033401','00182267901', |
|  | '00182267989','00339654512','00555083504','00615455729','00781038707','51079091620','59772038707','00056017401', |
|  | '00056017470','00056017475','00093172001','00378881001','00378881010','00406205901','00555083502','00832121900', |
|  | '00832121901','00832121950','00832121989','15330010801','21695080130','31722033501','43353005430','43353049130', |
|  | '43353057809','43353057830','51138006230','51138018730','51138020330','51138049010','51138049030','51672403501', |
|  | '51672403503','53217000100','53217000130','53217000160','53217000190','54569642700','54868245400','54868245401', |
|  | '54868245402','54868525800','55887056730','55887056760','55887056790','57237012701','57237012799','58016069700', |
|  | '58016069730','58016069760','58016069790','58118403503','58118403506','58118403509','60429079201','60429079230', |
|  | '60429079245','60429079277','63187075010','63187075030','65162076910','65162076911','66105052310','66267062800', |
|  | '68258606803','68258906401','68382005901','76282033501','00590032435','00590032496','49452813601','49452813602', |
|  | '51927247100','38779047405','38779047410','38779047425','38779047504','38779047505' |
| Pradaxa | '00597010754','00597010760','00597014954','00597014960','00597035509','00597035556','00597035561','00597010854', |
|  | '00597010860','00597013554','00597013560','00597036055','00597036082','21695089960','54569627600' |
| Eliquis | '00003089321','00003089331','50090143600','54569651300','00003089421','00003089431','00003089470','50090143700', |
|  | '54569651400' |
| Xarelto | '50458058010','50458058030','50458058090','50458057810','50458057830','50458057890','42254037601','50458057910', |
|  | '50458057930','50458057989','50458057990' |

**Suppl. table S2** Details of input and output medical conditions

| **Comorbidity Condition** | **ICD 10 Code Value** |
| --- | --- |
| Congestive Heart Failure Non-hypertensive | 'I0981','I501','I5020','I5021','I5022','I5023','I5030','I5031','I5032','I5033','I5040','I5041', |
|  | 'I5042','I5043','I50810','I50811','I50812','I50813','I50814','I5082','I5083','I5084','I5089','I509' |
| Essential Hypertension | 'I10' |
| Diabetes Mellitus 1 & 2 | 'E1010','E1011','E1021','E1022','E1029','E10311','E10319','E10321','E103211','E103212','E103213','E103219', |
|  | 'E10329','E103291','E103292','E103293','E103299','E10331','E103311','E103312','E103313','E103319','E10339','E103391', |
|  | 'E103392','E103393','E103399','E10341','E103411','E103412','E103413','E103419','E10349','E103491','E103492','E103493', |
|  | 'E103499','E10351','E103511','E103512','E103513','E103519','E103521','E103522','E103523','E103529','E103531','E103532', |
|  | 'E103533','E103539','E103541','E103542','E103543','E103549','E103551','E103552','E103553','E103559', |
|  | 'E10359','E103591','E103592','E103593','E103599','E1036','E1037X1','E1037X2','E1037X3','E1037X9', |
|  | 'E1039','E1040','E1041','E1042','E1043','E1044','E1049','E1051','E1052','E1059','E10610','E10618', |
|  | 'E10620','E10621','E10622','E10628','E10630','E10638','E10641','E10649','E1065','E1069','E108','E1100', |
|  | 'E1101','E1110','E1111','E1121','E1122','E1129','E11311','E11319','E11321','E113211','E113212','E113213','E113219','E11329', |
|  | 'E113291','E113292','E113293','E113299','E11331','E113311','E113312','E113313','E113319','E11339','E113391','E113392', |
|  | 'E113393','E113399','E11341','E113411','E113412','E113413','E113419','E11349','E113491','E113492','E113493','E113499', |
|  | 'E11351','E113511','E113512','E113513','E113519','E113521','E113522','E113523','E113529','E113531','E113532','E113533', |
|  | 'E113539','E113541','E113542','E113543','E113549','E113551','E113552','E113553','E113559','E11359','E113591','E113592', |
|  | 'E113593','E113599','E1136','E1137X1','E1137X2','E1137X3','E1137X9','E1139','E1140','E1141','E1142','E1143','E1144','E1149', |
|  | 'E1151','E1152','E1159','E11610','E11618','E11620','E11621','E11622','E11628','E11630','E11638','E11641','E11649','E1165','E1169','E118' |
| Ischemic Stroke | 'I6300','I63011','I63012','I63013','I63019','I6302','I63031','I63032','I63033','I63039','I6309','I6310','I63111','I63112','I63113','I63119', |
|  | 'I6312','I63131','I63132','I63133','I63139','I6319','I6320','I63211','I63212','I63213','I63219','I6322','I63231','I63232','I63233','I63239', |
|  | 'I6329','I6330','I63311','I63312','I63313','I63319','I63321','I63322','I63323','I63329','I63331','I63332','I63333','I63339','I63341','I63342', |
|  | 'I63343','I63349','I6339','I6340','I63411','I63412','I63413','I63419','I63421','I63422','I63423','I63429','I63431','I63432','I63433','I63439', |
|  | 'I63441','I63442','I63443','I63449','I6349','I6350','I63511','I63512','I63513','I63519','I63521','I63522','I63523','I63529','I63531','I63532', |
|  | 'I63533','I63539','I63541','I63542','I63543','I63549','I6359','I636','I638','I6381','I6389','I639' |
| Transient Ischemic Attack | 'G450','G451','G452','G453','G454','G458','G459' |
| Thrombo-Embolic Event | 'I7401','I7409','I7410','I7411','I7419','I742','I743','I744','I745','I748','I749', |
|  | 'I75011','I75012','I75013','I75019','I75021','I75022','I75023','I75029','I7581','I7589' |
| Acute Myocardial Infarction | I2101','I2102','I2109','I2111','I2119','I2121','I2129','I213','I214','I219','I21A1','I21A9','I220','I221','I222','I228','I229','I252' |
| Peripheral Artery Disease | I700','I701','I70201','I70202','I70203','I70208','I70209','I70211','I70212','I70213','I70218','I70219', |
|  | 'I70221','I70222','I70223','I70228','I70229','I70231','I70232','I70233','I70234','I70235','I70238','I70239', |
|  | 'I70241','I70242','I70243','I70244','I70245','I70248','I70249','I7025','I70291','I70292','I70293','I70298', |
|  | 'I70299','I708','I7090','I7091','I7092','I739','K550','K55011','K55012','K55019','K55021','K55022','K55029','K55031', |
|  | 'K55032','K55039','K55041','K55042','K55049','K55051','K55052','K55059','K55061','K55062','K55069','K551','K558','K559' |
| Valvular Disease | 'I050','I051','I052','I058','I059','I060','I061','I062','I068','I069','I070','I071','I072','I078','I079','I080', |
|  | 'I081','I082','I083','I088','I089','I091','I0989','I340','I341','I342','I348','I349','I350','I351','I352','I358', |
|  | 'I359','I360','I361','I362','I368','I369','I370','I371','I372','I378','I379' |
| Coronary Artery Disease | 'I2510','I25110','I25111','I25118','I25119','I25750','I25751','I25758','I25759','I25811' |
| Obstructive Sleep Apnea | 'G4730' |
| Chronic Kidney Disease | 'N181','N182','N183','N184','N185','N186','N189' |
| Chronic Obstructive Pulmonary Disease/Bronchiectasis | J40','J410','J411','J418','J42','J430','J431','J432','J438','J439','J440','J441','J449','J470','J471','J479' |
| Major Bleeding | 'H31301','H31302','H31303','H31309','H31311','H31312','H31313','H31319','H31411','H31412','H31413','H31419', |
|  | 'H3560','H3561','H3562','H3563', 'H35731','H35732','H35733','H35739', 'H4310','H4311''H4312','H4313', 'H47021', |
|  | 'H47022','H47023','H47029','I6000','I6001','I6002','I6010','I6011','I6012','I602','I6020','I6021','I6022', |
|  | 'I6030','I6031','I6032','I604','I6050','I6051','I6052','I606','I607','I608','I609','I610','I611','I612','I613', |
|  | 'I614','I615','I616','I618','I619','I6200','I6201','I6202','I6203','I621','I629','I6900','I6901','I69010','I69011', |
|  | 'I69012','I69013','I69014','I69015','I69018','I69019','I69020','I69021','I69022','I69023','I69028','I69031','I69032', |
|  | 'I69033','I69034','I69039','I69041','I69042','I69043','I69044','I69049','I69051','I69052','I69053','I69054','I69059', |
|  | 'I69061','I69062','I69063','I69064','I69065','I69069','I69090','I69091','I69092','I69093','I69098','I6910', |
|  | 'I6911','I69110','I69111','I69112','I69113','I69114','I69115','I69118','I69119','I69120','I69121','I69122','I69123', |
|  | 'I69128','I69131','I69132','I69133','I69134','I69139','I69141','I69142','I69143','I69144','I69149','I69151','I69152', |
|  | 'I69153','I69154','I69159','I69161','I69162','I69163','I69164','I69165','I69169','I69190','I69191','I69192','I69193', |
|  | 'I69198','I6920','I6921','I69210','I69211','I69212','I69213','I69214','I69215','I69218','I69219','I69220','I69221', |
|  | 'I69222','I69223','I69228','I69231','I69232','I69233','I69234','I69239','I69241','I69242','I69243','I69244','I69249', |
|  | 'I69251','I69252','I69253','I69254','I69259','I69261','I69262','I69263','I69264','I69265','I69269','I69290','I69291', |
|  | 'I69292','I69293','I69298','D68311','D68312','D68318','D6832','D698','D699','H1130','H1131','H1132','H1133', |
|  | 'H3120','H2100','H2101','H2102','H2103','H9220','H9221','H9222','H9223','I312','I850','I8501','I8511','K226', |
|  | 'K250','K251','K252','K254','K255','K256','K260','K261','K262','K264','K265', 'K266','K270','K271','K272','K274', |
|  | 'K275','K276','K280','K281','K282','K284','K285','K286','K290','K2901', 'K2921','K2931','K2941','K2951','K2961', |
|  | 'K2971','K2981','K2991','K920','K921','K922','I9820','I983','K2210', 'K2212','K2214','K2216','K6380','K3180','K5520', |
|  | 'K625','K922' |
| Alcohol Use | 'F1010','F1011','F10120','F10121','F10129','F1014','F10150','F10151','F10159','F10180','F10181','F10182', |
|  | 'F10188','F1019','F1020','F1021','F10220','F10221','F10229','F10230','F10231','F10232','F10239','F1024', |
|  | 'F10250','F10251','F10259','F1026','F1027','F10280','F10281','F10282','F10288','F1029','F10920','F10921', |
|  | 'F10929','F1094','F10950','F10951','F10959','F1096','F1097','F10980','F10981','F10982','F10988','F1099' |
| Alcohol Disorder | 'G621','I426','K2920','K2921','K700','K7010','K7011','K702','K7030','K7031','K7040','K709' |
| Inflammatory Disease | 'M450','M451','M452','M453','M454','M455','M456','M457','M458','M459','M488X1','M488X2','M488X3', |
|  | 'M488X4','M488X5','M488X6','M488X7','M488X8','M488X9','M1000','M10011','M10012','M10019','M10021','M10022', |
|  | 'M10029','M10031','M10032','M10039','M10041','M10042','M10049','M10051','M10052','M10059','M10061','M10062', |
|  | 'M10069','M10071','M10072','M10079','M1008','M1009','M1010','M10111','M10112','M10119','M10121','M10122', |
|  | 'M10129','M10131','M10132','M10139','M10141','M10142','M10149','M10151','M10152','M10159','M10161','M10162', |
|  | 'M10169','M10171','M10172','M10179','M1018','M1019','M1020','M10211','M10212','M10219','M10221','M10222', |
|  | 'M10229','M10231','M10232','M10239','M10241','M10242','M10249','M10251','M10252','M10259','M10261','M10262', |
|  | 'M10269','M10271','M10272','M10279','M1028','M1029','M1030','M10311','M10312','M10319','M10321','M10322', |
|  | 'M10329','M10331','M10332','M10339','M10341','M10342','M10349','M10351','M10352','M10359','M10361','M10362', |
|  | 'M10369','M10371','M10372','M10379','M1038','M1039','M1040','M10411','M10412','M10419','M10421','M10422', |
|  | 'M10429','M10431','M10432','M10439','M10441','M10442','M10449','M10451','M10452','M10459','M10461','M10462', |
|  | 'M10469','M10471','M10472','M10479','M1048','M1049','M109','M320','M3210','M3211','M3212','M3213','M3214', |
|  | 'M3215','M3219','M328','M329','M0540','M05411','M05412','M05419','M05421','M05422','M05429','M05431','M05432', |
|  | 'M05439','M05441','M05442','M05449','M05451','M05452','M05459','M05461','M05462','M05469','M05471','M05472', |
|  | 'M05479','M0549','M0550','M05511','M05512','M05519','M05521','M05522','M05529','M05531','M05532','M05539','M05541', |
|  | 'M05542','M05549','M05551','M05552','M05559','M05561','M05562','M05569','M05571','M05572','M05579','M0559','M0560', |
|  | 'M05611','M05612','M05619','M05621','M05622','M05629','M05631','M05632','M05639','M05641','M05642','M05649','M05651', |
|  | 'M05652','M05659','M05661','M05662','M05669','M05671','M05672','M05679','M0569','M0570','M05711','M05712','M05719', |
|  | 'M05721','M05722','M05729','M05731','M05732','M05739','M05741','M05742','M05749','M05751','M05752','M05759','M05761', |
|  | 'M05762','M05769','M05771','M05772','M05779','M0579','M0580','M05811','M05812','M05819','M05821','M05822','M05829', |
|  | 'M05831','M05832','M05839','M05841','M05842','M05849','M05851','M05852','M05859','M05861','M05862','M05869','M05871', |
|  | 'M05872','M05879','M0589','M059','M0600','M06011','M06012','M06019','M06021','M06022','M06029','M06031','M06032', |
|  | 'M06039','M06041','M06042','M06049','M06051','M06052','M06059','M06061','M06062','M06069','M06071','M06072','M06079', |
|  | 'M0608','M0609','M061','M0620','M06211','M06212','M06219','M06221','M06222','M06229','M06231','M06232','M06239','M06241', |
|  | 'M06242','M06249','M06251','M06252','M06259','M06261','M06262','M06269','M06271','M06272','M06279','M0628','M0629', |
|  | 'M0630','M06311','M06312','M06319','M06321','M06322','M06329','M06331','M06332','M06339','M06341','M06342','M06349', |
|  | 'M06351','M06352','M06359','M06361','M06362','M06369','M06371','M06372','M06379','M0638','M0639','M064','M0680', |
|  | 'M06811','M06812','M06819','M06821','M06822','M06829','M06831','M06832','M06839','M06841','M06842','M06849', |
|  | 'M06851','M06852','M06859','M06861','M06862','M06869','M06871','M06872','M06879','M0688','M0689','M069' |
| Lipid Disorders | 'E780','E7800','E7801','E781','E782','E783','E784','E7841','E7849','E785' |

**Suppl. table S3** Description of machine learning algorithms

| **Machine learning algorithm** | **Description** |
| --- | --- |
| Logistic regression | The machine learning based logistic regression algorithm included main effects, interaction terms and polynomial effects, with the model selection based on the stepwise method. Only quadratic terms were included in the polynomial formulation to ensure proper conversion in a timely fashion of the optimization algorithm for logistic regression from numerical analysis perspective, given the sheer volume of sample size, large number of multi-morbid conditions as well as the consideration of different types of interactions and polynomial terms. |
| Neural network | The neural network algorithm used a multilayer perceptron architecture with direct connection. Classically, the multiceptron is a feed-forward multilayer network architecture composed of several layers of neurons, an input layer, an output layer, and several hidden layers. For most problems, a network with one hidden layer is used, but in this investigation, five hidden layers were deemed appropriate to handle the model complexity in this study. A single run of the neural network algorithm for a given clinical outcome was much more efficient than a logistic regression algorithm. |
| Decision tree | The decision tree was produced by algorithms that identify various ways of splitting a data set into branch-like segments. These segments form an inverted decision tree that originates with a root node at the top of the tree. The object of analysis is reflected in this root node as a simple, one dimensional display in the decision tree interface. In this algorithm, we allowed the splitting rule for a maximum depth up to 10 generations of split. The probability of chi-square was used for the evaluation of nominal targets. With respect to a node, the leaf size was set at 8, with up to 4 surrogate rules for non-leaf nodes. |
| Gradient boosting | Gradient boosting uses a partitioning algorithm to search for an optimal partition of the data for a single target variable. It resamples the analysis data several times to generate results that form a weighted average of the resampled data set. Tree boosting creates a series of decision trees that form a single predictive model. Like decision trees, boosting makes no assumptions about the distribution of the data. Boosting is less prone to overfit the data than a single decision tree. If a decision tree fits the data fairly well, then boosting often improves the fit. It should be noted that the maximum depth for splitting rules was set at 10. Also, the number of surrogate rules was established at 2. |

**Suppl. fig 1** Clinical utility for multimorbid index/AF status/medicare status model and logistic regression machine learning-based algorithm

a b

**Suppl. fig 2** Importance of variables for the a) gradient boosting, b) neural network and c) logistic regression algorithms

c

**Suppl. fig 2** Cont’d
